# Application of Genomic Offsets to Inform Freshwater Fisheries Management Under Climate Change

**DOI:** 10.1111/eva.70149

**Published:** 2025-08-27

**Authors:** Anna S. Jacquemart, Anna Tigano, Marika Kirstin Gale, Tyler Weir, Hillary G. M. Ward, Carmen M. Wong, Erika J. Eliason, Kristina M. Miller, Scott G. Hinch, Michael A. Russello

**Affiliations:** ^1^ Department of Biology The University of British Columbia Okanagan Kelowna British Columbia Canada; ^2^ Pacific Biological Station, Fisheries and Oceans Canada Nanaimo British Columbia Canada; ^3^ Freshwater Fisheries Society of BC Victoria British Columbia Canada; ^4^ Aquatic Ecosystems Branch, British Columbia Ministry of Water Land and Resource Stewardship Victoria British Columbia Canada; ^5^ Resource Management Branch, British Columbia Ministry of Water Land and Resource Stewardship Penticton British Columbia Canada; ^6^ Yukon Field Unit, Parks Canada Whitehorse Yukon Territories Canada; ^7^ Department of Ecology, Evolution, and Marine Biology University of California Santa Barbara Santa Barbara California USA; ^8^ Pacific Science Enterprise Center Fisheries and Oceans Canada West Vancouver British Columbia Canada; ^9^ Department of Forest and Conservation Sciences, Pacific Salmon Ecology and Conservation Laboratory The University of British Columbia Vancouver British Columbia Canada

**Keywords:** adaptive potential, donor and recipient importance, genomic vulnerability, genotyping‐by‐sequencing, gradient forest, pacific salmon

## Abstract

Genomic tools are becoming increasingly necessary for mitigating biodiversity loss and guiding management decisions in the context of climate change. Freshwater fish species are particularly susceptible to the impacts of changing environments, including kokanee, the resident form of sockeye salmon ( *Oncorhynchus nerka* ), which has already been negatively impacted by increases in extreme temperature throughout its distribution. A previous study using whole genome resequencing of wild kokanee stocks identified 1412 environmentally associated SNPs and demonstrated genomic offset, a measure of climate vulnerability, to be significantly correlated with higher increases in extreme warm temperatures across much of the species' range in western Canada. Here, we aimed to operationalize this information for fisheries management by first developing a Genotyping‐in‐Thousands by sequencing (GT‐seq) panel populated exclusively with environment associated SNPs. We then evaluated the robustness of the GT‐seq panel relative to the signal in the whole genome resequencing baseline and demonstrated a novel application of donor and recipient importance (DI/RI) analysis to inform recreational fisheries stocking decisions. We found that a reduced GT‐seq panel of 616 SNPs exhibited a significant positive correlation with those calculated from the full set of 1412 SNPs across the climate change scenarios tested; similar results were obtained when adding new reference populations not included in the original whole genome resequencing baseline. The DI/RI analysis revealed clear spatial trends, with populations situated in the warmest regions of southern interior British Columbia (Canada) having the highest probability for successful translocations to different recipient locations to the north. Similarly, candidate recipient lakes for stocking at the center of the distribution had higher recipient importance values than those located towards the eastern and western range peripheries. Although further refinement is required, pairing targeted genotyping with genomic offset and DI/RI predictions holds great promise for informing freshwater fisheries management moving forward.

## 1 Introduction

Freshwater systems have experienced steeper vertebrate population declines than in the terrestrial and marine environments, with modern extinction rates over 900 times greater than background levels (Burkhead 2012; Ceballos et al. 2015; Dudgeon 2019; Krabbenhoft et al. 2020; Lynch et al. 2016, 2023; Myers et al. 2017; Reid et al. 2019). In North America alone, at least 39 freshwater fish species have gone extinct since 1900 (Burkhead 2012). Climate change is considered the main stressor to many freshwater fish, threatening approximately 50% of all extant species (Krabbenhoft et al. 2020; Reid et al. 2019; Sharma et al. 2011). Moreover, other anthropogenic‐mediated threats such as emergence of diseases (Borgwardt et al. 2020), habitat degradation (Radinger et al. 2017), invasive species (Sharma et al. 2011), and pollution (Arthington et al. 2016; Reid et al. 2019) can compound the effects of climate change (and vice versa) on freshwater fishes, further increasing their vulnerability. Some freshwater fish species are particularly sensitive to environmental change due to limited range and dispersal potential, which can lead to a higher risk of extirpation or extinction compared to marine fish species (Arthington et al. 2016; Chu et al. 2005; Comte and Grenouillet 2013; Dudgeon 2019; Krabbenhoft et al. 2020; Myers et al. 2017; Radinger et al. 2017).

Genetic and genomic tools have long been employed in fisheries management, yet have only recently been used to predict vulnerability to climate change and to develop strategies for mitigating its effects (Layton et al. 2021; Lynch et al. 2023; Tigano et al. 2024). Initial studies have shown that an understanding of genomic variation associated with local adaptation can assist in projecting the evolutionary change necessary for a species to track changes in the environment (Chen et al. 2022; Colella et al. 2020; Layton et al. 2021). Genomic offset (also referred to as genetic offset, genomic vulnerability or risk of non‐adaptedness) represents the distance between current and required genomic composition for adaptive loci under changing conditions and provides a relative measure of the evolutionary change necessary to avoid maladaptation and deleterious fitness effects (Fitzpatrick et al. 2021; Fitzpatrick and Keller 2015; Láruson et al. 2022; Layton et al. 2021; Layton and Bradbury 2022; Rellstab et al. 2021; but see Lind and Lotterhos 2025; Lotterhos 2024). This approach can help identify populations most at risk for fitness decline under different climate change scenarios (Láruson et al. 2022; Razgour et al. 2019; Rellstab et al. 2021).

Genomic data have also been used to inform assisted migration of targeted individuals for a range of conservation applications, including to reduce extinction risk of populations susceptible to climate change (Chen et al. 2022; Forester et al. 2022; Razgour et al. 2019; Ste‐Marie et al. 2011). This management strategy relies on the establishment of criteria for designating source and recipient populations to maximize overall genetic diversity or introduce locally adaptive alleles (Chen et al. 2022; Rellstab et al. 2021). Although not yet applied in a fisheries context, genomic offset has been employed in the forestry sector to help identify candidate recipient and source populations to inform assisted migration practices (Chen et al. 2022; Lachmuth, Capblancq, Keller, and Fitzpatrick 2023; Lachmuth, Capblancq, Prakash, et al. 2023; Láruson et al. 2022; Razgour et al. 2019). For example, measures of Donor/Recipient Importance (DRI) have been developed based on standardized genomic offset values to evaluate transferability of individuals from one source location to a recipient location (Lachmuth, Capblancq, Keller, and Fitzpatrick 2023; Lachmuth, Capblancq, Prakash, et al. 2023). Donor Importance (DI) is defined as the proportion of recipient locations to which individuals from a specific population could be transferred without exceeding a tolerable threshold level of genomic offset (Lachmuth, Capblancq, Prakash, et al. 2023). Likewise, Recipient Importance (RI) is defined as the proportion of donor populations that could be transferred to a specific recipient location without exceeding a tolerable threshold level of offset (Lachmuth, Capblancq, Prakash, et al. 2023). The Donor/Recipient Importance concept was initially developed in a study aimed at identifying the best candidate populations for translocation and in situ conservation of red spruce ( *Picea rubens* ) from northeast USA and eastern Canada (Lachmuth, Capblancq, Keller, and Fitzpatrick 2023; Lachmuth, Capblancq, Prakash, et al. 2023). The use of these metrics could potentially be extended to freshwater fisheries management to identify optimal source stocks for specific recipient locations.

Kokanee, the freshwater resident form of sockeye salmon ( *Oncorhynchus nerka* ), is mainly present across the Pacific Northwest region of North America, although populations do exist elsewhere across the full Pan‐Pacific species distribution (Quinn 2018). In North America, kokanee represents a food, social, and ceremonial fishery for First Nations, a preferred year‐round source of sustenance for non‐Indigenous communities, and a key species for economically important recreational fisheries (Bailey and Sumaila 2012; Falke et al. 2015; Salisbury et al. 2023; Ward et al. 2019). Modeling studies based on various climate change scenarios predict a ~50% decline in suitable habitat for trout and other cold‐water specialists, such as kokanee, in the western United States by 2080 (Wenger et al. 2011). Moreover, experimental studies in sockeye salmon have highlighted the potential negative effects of climate change on embryo survival (Whitney et al. 2013) and swimming capabilities (Eliason et al. 2011). Indeed, rising water temperatures during the spawning migration of sockeye salmon in the Fraser River have resulted in high mortality events (Crossin et al. 2008; Macdonald et al. 2010; Martins et al. 2011). Across the kokanee range, there has been a notable increase in extreme climatic events and significantly elevated freshwater temperatures, with climate models predicting an increase in warmer air temperatures between 3.7°C and 11.2°C over the next 20–40 years (Islam et al. 2019; Tigano et al. 2024). As kokanee represents a species of strategic importance for supporting freshwater fisheries in western Canada, the need to ramp up hatchery production of stocks robust to changing environments is now viewed as a high priority by managers in government and non‐government agencies (Bailey and Sumaila 2012).

Genomic vulnerability to climate change has been assessed previously for kokanee in BC and Yukon using whole genome data (Tigano et al. 2024). A set of 1412 environment‐associated SNPs was identified through redundancy analysis (RDA) of genomic variation and bioclimatic variables related to extreme temperatures, precipitation seasonality, and precipitation levels, as well as pH (Tigano et al. 2024). Warmest temperatures showed the strongest association with genomic variation in this system, and genomic offset estimates were highly correlated with expected increases in extreme air temperatures (Tigano et al. 2024). For example, the southern interior of BC, which is predicted to experience the largest increases in maximum temperatures, had the highest offset estimates indicating a greater risk of maladaptation under climate change compared to regions with relatively lower increases in warmest temperature, such as in the northern part of the range or on Vancouver Island (Tigano et al. 2024). Those results provide an excellent baseline to develop a tool to estimate genomic vulnerability of wild stocks across the kokanee range, inform broodstock management, and guide targeted stocking that matches optimal wild donor stocks to selected recipient locations to support recreational fisheries.

Here, we tested an approach to operationalize genomic offset estimates for informing freshwater fisheries management, using kokanee as a case study. First, we developed a new genotyping‐in‐thousands by sequencing (GT‐seq; Campbell et al. 2015) panel populated exclusively with the environment‐associated SNPs identified in Tigano et al. (2024) from a large set of wild stock kokanee sampled across the Canadian range. We then tested the robustness of the new GT‐seq panel for estimating genomic offset relative to the whole‐genome data, and for adding new wild stock locations to the genomic offset baseline. Lastly, we used the empirical data at these targeted SNPs for candidate donor populations, in tandem with potential stocking locations, to demonstrate how Donor/Recipient Importance metrics could be used to inform kokanee fisheries management in the face of climate change. Our results demonstrate the potential for applying genomic offsets for informing regional and global fisheries management practices moving forward.

## 2 Methods

### 2.1 SNP Discovery and Panel Design

We used the initial set of 1412 environment‐associated SNPs identified in Tigano et al. (2024) (hereafter referred to as the “WGS‐1412” dataset; Table S1) for GT‐seq panel design. To test whether reduced subsets of SNPs could replicate estimates based on the original full set in the event of potential SNP fallout during panel optimization, we estimated genomic offset with 1200, 600, or 300 randomly selected SNPs following the methods used in Tigano et al. (2024). All analyses were performed using the R package *gradientforest* (Ellis et al. 2012; R Core Team 2021) and two different climate change scenarios for the 2041–2060 time period (RPC4.5 and RPC8.5). This analysis involves a regression tree approach utilizing machine learning, which generates nonlinear cumulative importance functions that describe allele turnover across environmental gradients (Ellis et al. 2012). This approach has emerged as a preferred option for genomic offset studies performed over environmental gradients (Ellis et al. 2012; Fitzpatrick and Keller 2015; Láruson et al. 2022; Schmidt and Russello 2025).

After having verified that smaller numbers of SNPs could replicate genomic offset estimates based on the WGS‐1412 dataset, 200 bp sequences (~100 bp on either side) flanking all environment‐associated SNPs (*n* = 1412) were pulled using vcf tools (Danecek et al. 2011) and sent to GTseek LLC for custom locus‐specific primer design. Loci were split between two panels after primer design to minimize primer interactions and SNP fallout during optimization.

### 2.2 GT‐Seq Test Library Preparation

We constructed GT‐seq test libraries from 83 DNA samples, 52 of which were previously included in the WGS study (Tigano et al. 2024), from 16 wild stock populations across BC and Yukon. Populations were defined by geographic location, all of which were genetically distinct (Tigano et al. 2024). This design allowed us to evaluate genotyping discordance between WGS and GT‐seq data. We also included eight technical replicates to evaluate within GT‐seq genotyping discordance. Library preparation followed the protocol described in Campbell et al. (2015), with modifications outlined in Schmidt et al. (2020). PCR2 products were quantified using a Qubit Flex Fluorometer (Invitrogen) and normalized to 7 ng/μL before pooling 5 μL of each normalized product. The pooled library was purified using a MinElute PCR Purification Kit (Qiagen) and eluted into 24 μL of distilled water. We evaluated the size fragment distribution of the library using a D1000 ScreenTape on an Agilent 2200 TapeStation. Libraries were paired‐end (150 bp) sequenced using an Illumina MiniSeq.

### 2.3 GT‐Seq Panel Optimization and Genotyping

Raw reads were demultiplexed and genotyped following the GT‐seq pipeline (https://github.com/GTseq/GTseq‐Pipeline; Campbell et al. 2015). For each round of optimization, we removed: (1) loci accounting for > 1% of total raw reads; (2) observed primer dimers; and (3) loci with no read counts. After multiple rounds of optimization, we removed individuals and then SNPs with > 35% missing data. Genotyping error was then calculated as the discordance of genotypes between replicated individuals within GT‐seq libraries as well as for individuals genotyped using both GT‐seq and WGS from Tigano et al. (2024) (https://github.com/bsjodin/genoerrorcalc). We detected and removed a subset of SNPs (*n* = 89) that exhibited discordance > 50% between the GT‐seq and WGS datasets in order to minimize bias associated with the repetitive nature of the sockeye salmon genome (Christensen et al. 2020) and differences between data collection methods.

The optimized GT‐seq panel was used to genotype 248 new individuals from the same 16 lakes in the WGS baseline to test whether sampling different individuals from the same stocks would produce similar genomic offset estimates (Table 1). In addition, to evaluate the effects of adding previously unsampled wild stock populations to relative genomic offset estimates, we included 42 samples from three additional lakes in BC (Alouette, Tatuk and Duncan Lakes; Table 1) (see below). The Alouette and Duncan Lake populations have been previously demonstrated to be genetically distinct (Lemay and Russello 2012; Samad‐zada, Nakayama, and Russello 2021; Samad‐zada, van Poorten, et al. 2021); Tatuk has not been previously analyzed, but it is located in an extremely isolated location in central BC. DNA was extracted from all new tissue samples using a standard Chelex‐based protocol (Walsh et al. 2013) and individuals were genotyped following the same methods as described above. This final GT‐seq dataset containing only unique individuals not included in the original WGS baseline is hereafter referred to as the “GTseq‐616” dataset (Table S1).

**TABLE 1:** Location and retained sample size for populations included in this study.

Population	Latitude	Longitude	*N*_GTseq_ ^a^	*N*_WGS_ ^b^
Alouette^c^	−122.25	49.20	7 (0)	0
Anderson	−122.39	50.65	17 (9)	12
Arctic	−121.69	54.42	9 (2)	11
Arrow	−117.96	50.14	9 (1)	8
Bonaparte	−120.54	51.26	16 (3)	5
Christina	−118.26	49.14	20 (2)	12
Cowichan	−124.29	48.88	16 (3)	6
Duncan^c^	−126.47	57.58	27 (0)	0
East Barriere	−119.80	51.27	18 (1)	12
Kalamalka	−119.34	50.17	18 (3)	23
Kootenay	−116.80	49.49	14 (0)	9
Nicola	−120.53	50.16	19 (3)	9
Okanagan	−119.47	50.01	18 (11)	24
Puntzi	−124.02	52.19	12 (2)	11
Shawningan	−123.64	48.63	10 (2)	10
Sockeye	−137.62	60.50	17 (3)	10
Tatuk^c^	−124.23	53.52	8 (0)	0
Tchesinkut	−125.64	54.09	18 (2)	11
Wood	−119.40	50.06	18 (3)	21

^a^ Numbers in parentheses indicate retained samples repeated from WGS dataset (e.g., for Anderson, a total of 17 samples were genotyped via GTseq, 9 of which were included in the original WGS analysis).

^b^ Data from Tigano et al. (2024).

^c^ New populations included in Expanded‐616 and DRI‐616 datasets.

### 2.4 Genomic Offset

To calculate genomic offset based on the GT‐seq data, we used the Gradient Forest method as in the initial simulations. Gradient Forest models were parameterized using GTseq‐616, the four WorldClim bioclimatic variables significantly associated with the putatively adaptive SNPs included in the panel following Tigano et al. (2024) (bio5, maximum temperature of warmest month; bio6, minimum temperature of coldest month; bio15, precipitation seasonality; bio16, precipitation of wettest quarter), and 500 regression trees per SNP (Ellis et al. 2012). Genomic offset values for each population were calculated as the Euclidean distance between current and future genetic importance values under different climate change scenarios (RCP4.5 and RCP8.5). Raw genomic offsets were standardized following the approach outlined in Lachmuth, Capblancq, Keller, and Fitzpatrick (2023), and standardized offsets were scaled for results visualization.

To assess concordance between genomic offset estimates, we first compared values from WGS‐1412 with a subsample of the same dataset containing only the SNPs retained after panel optimization (hereafter referred to as the “WGS‐616” dataset; Table S1) by fitting linear regression models using the *lm* function in R. Secondly, using the same linear regression method, we compared genomic offset estimates from the WGS‐1412 and GTseq‐616 datasets, the latter composed exclusively of new individuals genotyped from each location. We also tested the impacts to relative genomic offset estimates of adding new wild stock populations (Alouette, Tatuk and Duncan Lake) genotyped via GT‐seq to the WGS‐616 baseline (hereafter referred to as the “Expanded‐616”; Table S1). Genomic offset was then re‐calculated for all populations, and linear regression was again used to compare values relative to those from the original WGS‐1412 dataset.

### 2.5 Donor/Recipient Importance

Given the strong correlation between genomic offset values calculated across data collection approaches (see Results), a final dataset was compiled that included all unique individuals genotyped using either WGS or GT‐seq in order to maximize sample sizes (hereafter referred to as the “DRI‐616”; Table S1). Genomic offset values were estimated for each potential wild stock donor population and used to calculate DI and RI between current kokanee populations and potential recipient lakes (Lachmuth, Capblancq, Prakash, et al. 2023). Recipient lakes were identified by the Freshwater Fisheries Society of British Columbia as locations for current or future stocking, with no documented history of wild stock kokanee. We calculated temporal genomic offset values for potential wild stock donor populations using the current climate conditions at the original location and the projected climatic conditions at each potential recipient location according to the two climate change scenarios used previously (2041–2060, RCP4.5 and RCP8.5) with the *offsetEnsembleR* package in R (Lachmuth, Capblancq, Keller, and Fitzpatrick 2023). Spatio‐temporal offset values were standardized by rescaling offset estimates from 0 to 1 relative to contemporary spatial offset estimates to allow for further analysis. The threshold of biologically tolerable offset to calculate DI and RI was set to one standard deviation of calculated standardized offsets following Lachmuth, Capblancq, Keller, and Fitzpatrick (2023).

## 3 Results

### 3.1 Panel Optimization

Genomic offset values calculated from all random subsets of 1200, 600, and 300 SNPs showed strong linear relationships with values calculated from WGS‐1412 (mean *R* ^2^ = 0.96, *p* < 0.001 across all subsets; Figure S1). Of the original 1412 SNPs, primers were successfully designed for 1161 SNPs and split into two panels to minimize primer interactions (panel 1, *n* = 602; panel 2, *n* = 559). The initial test library was composed of all 1161 SNPs (bio5 = 428, bio6 = 280, bio15 = 283, bio16 = 17, and pH = 153). After four rounds of optimization, 616 SNPs (mean read depth = 116.85), 44 of 52 replicate samples between WGS and GT‐seq, and six of eight technical replicates were retained. Mean genotyping error among replicates within the GT‐seq library was 0.22%, while median genotyping discordance among retained individuals genotyped using both GT‐seq and WGS was 9.23%. Following genotyping with the finalized GT‐seq panel and subsequent filtering, we retained 199 new individuals (i.e., not included in the WGS baseline) distributed across the 16 original sites as well as 42 individuals from three new locations (Table 1).

### 3.2 Genomic Offset

**FIGURE 1:**
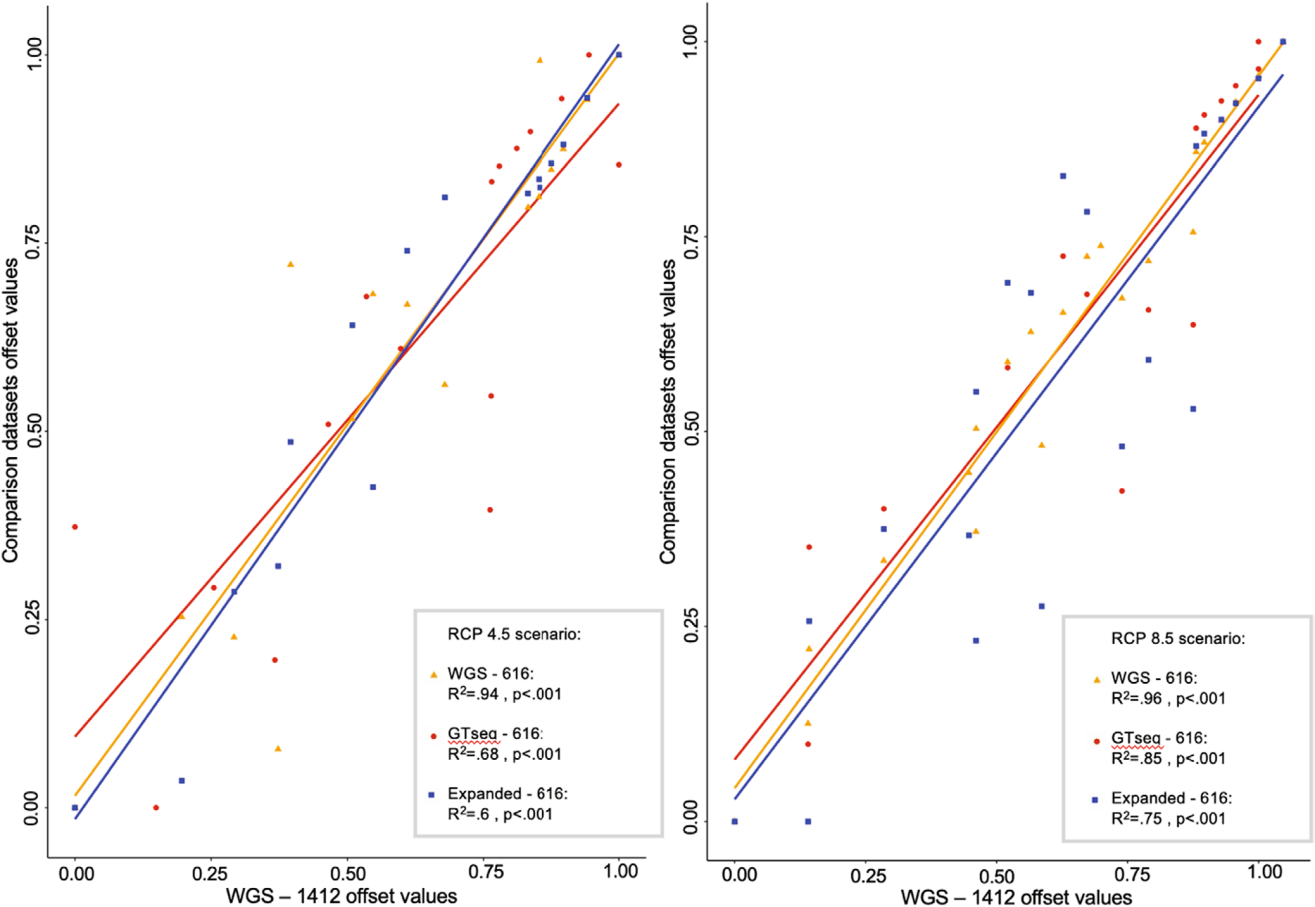
Linear regression analysis of genomic offset values calculated for the WGS‐1412 dataset relative to three other datasets (WGS‐616, GTseq‐616, and Expanded‐616) under the RCP4.5 (left) and RCP8.5 (right) scenarios.

**FIGURE 2:**
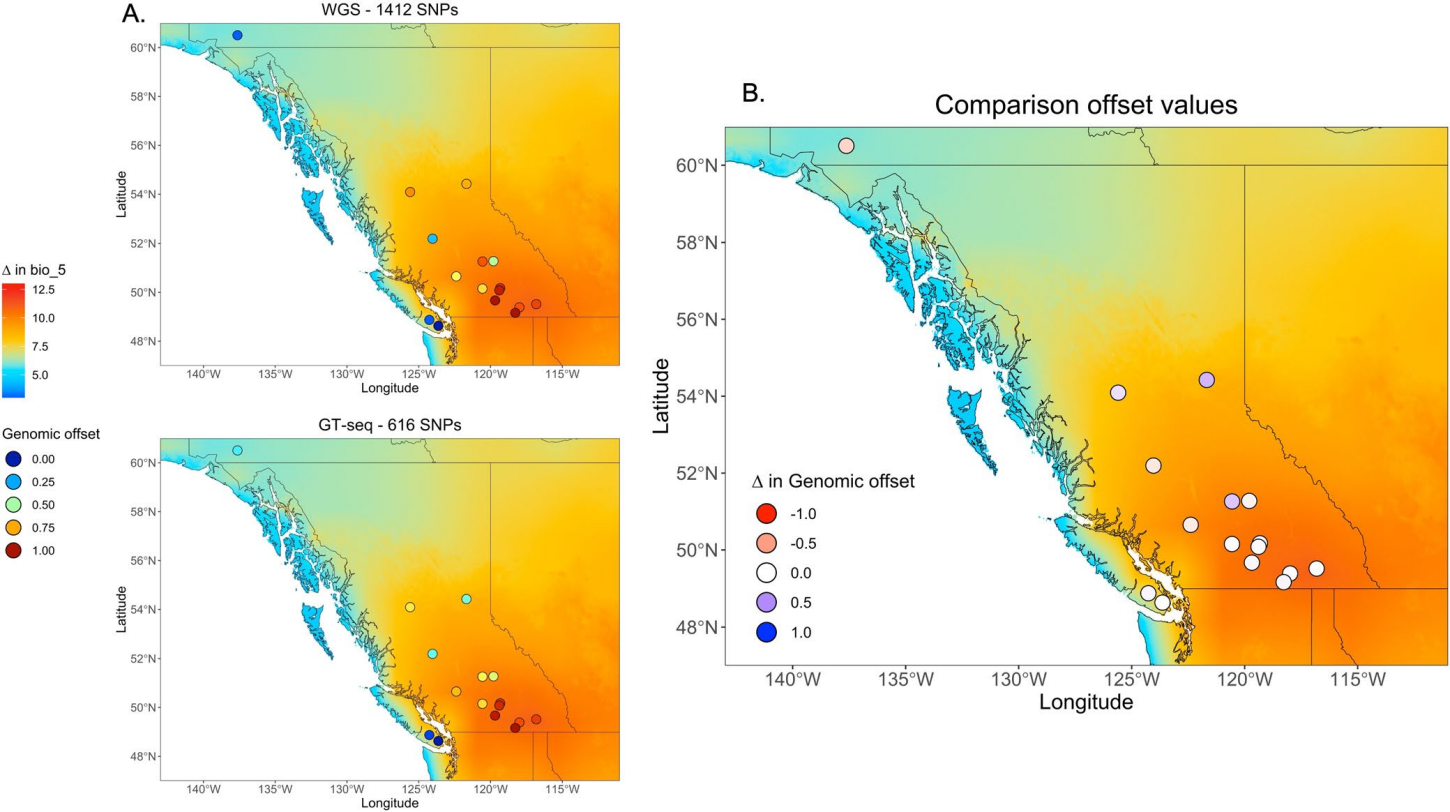
Maps of the spatial distribution and relative difference of genomic offset values calculated from different datasets under the RCP8.5 climate change scenario. (A) Spatial distribution of genomic offsets for the 16 wild stock lakes in BC and Yukon recalculated from the WGS dataset of Tigano et al. (2024) containing all 1412 environment‐associated SNPs (WGS‐1412; top) and based on GT‐seq data from new individuals genotyped from these same locations (GTseq‐616). (B) The relative change in genomic offsets between WGS‐1412 and GTseq‐616 at all 16 locations. The base map in all shows the difference in the warmest temperature of the warmest month (ΔT bio5) between current and predicted measures for 2041–2060 for the RCP8.5 climate change scenario.

Predicted genomic offset values estimated using the WGS‐616 dataset exhibited a significant positive correlation with those calculated from the WGS‐1412 dataset (RCP4.5: *R* ^2^ = 0.94, *p* < 0.001; RCP8.5: *R* ^2^ = 0.96, *p* < 0.001; Figure 1 and Tables S2 and S3). Likewise, predicted genomic offset values at each location calculated from the GTseq‐616 dataset that were composed entirely of individuals not included in the WGS baseline exhibited a strong, positive relationship with those calculated from WGS‐1412 (RCP4.5: *R* ^2^ = 0.68, *p* < 0.001; RCP8.5: *R* ^2^ = 0.85, *p* < 0.001; Figures 1 and 2; Tables S2 and S3; Figure S2). Similar results were obtained using the Expanded‐616 dataset that added three new populations (Alouette, Tatuk and Duncan) not included in the original WGS‐1412 dataset (RCP4.5: *R* ^2^ = 0.6, *p* < 0.001; RCP8.5: *R* ^2^ = 0.75, *p* < 0.001) (Figure 1; Tables S2 and S3). Overall, genomic offsets calculated from the GTseq‐616 and WGS‐1412 datasets exhibited similar spatial distributions of values across BC and Yukon (Figure 2A), with only marginal differences at the site level (Figure 2B).

### 3.3 Donor/Recipient Importance

**FIGURE 3:**
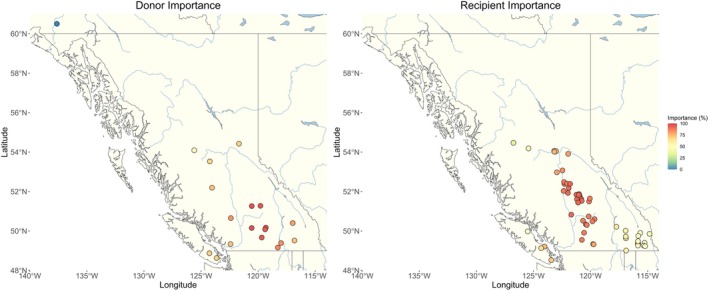
Donor Importance and Recipient Importance for 2041–2060 of the RPC8.5 climate change scenario. Donor Importance is shown for all 19 wild stock lakes (Table 1) that could be candidate sources for the stocking program. Recipient Importance is shown for lakes without wild kokanee that are either currently stocked or candidates for future stocking as part of the recreational fisheries management program in BC.

Donor importance values calculated from the DRI‐616 dataset (Tables S1 and S4) ranged from 0.00% to 96.55%. Populations in the south of BC showed the highest DI under both the RCP4.5 and RCP8.5 scenarios, with the largest values exhibited by Bonaparte and Nicola (96.55%), and Kalamalka and Okanagan (94.82%). Populations at the northern and western range peripheries showed the lowest DI (Figure 3 and Table S4), with Sockeye (0.00%), the northernmost population in the dataset, having the smallest value.

Recipient importance results followed a similar pattern under both tested climate change scenarios, with lakes at the center of the distribution of potential stocking locations exhibiting higher RI than lakes at the range periphery (Figure 3 and Table S5). Locations with the highest RI were Deka, Hathaway, Sulphurous, Bridge, Horse, and Timothy (91.66%). There were two clusters of lakes exhibiting lower RI, one at the western edge of the range on Vancouver Island and the other at the most eastern periphery of the range, with the lowest RI values exhibited by Beavertail, Prospect, and Shelton (50%).

## 4 Discussion

Developing genomic tools to assist fisheries management efforts is becoming increasingly important in the face of climate change. A number of different methods and metrics, such as genomic vulnerability and donor/recipient importance (DI/RI), may hold promise for informing management decisions across multiple sectors (e.g., forestry, fisheries, wildlife), but are still in their infancy in terms of application. Here, we show how genomic data can be used for developing targeted SNP genotyping panels for estimating genomic vulnerability to climate change and demonstrate a novel application of DI/RI analysis for informing freshwater fisheries management.

### 4.1 GT‐Seq and Genomic Offset

Genomic offset results were effectively replicated between datasets, suggesting that a reduced GT‐seq panel can accurately reflect the signal of a larger set of environment‐associated SNPs originally identified using WGS data for the purposes of these applications. We initially found high collinearity between genomic offset values calculated from the baseline WGS‐1412 dataset and the WGS‐616 dataset (Figures 1 and 2). These results were largely mirrored when we used the GTseq‐616 dataset that was composed exclusively of new individuals from the same 16 populations present in the WGS baseline (Figures 1 and 2), providing important validation of this tool moving forward.

Our results further suggest that the GT‐seq panel can be used to expand the baseline as samples from new wild stock lakes become available. When adding in the Alouette, Tatuk, and Duncan populations, for which no WGS data were available (Expanded‐616), relative genomic offset calculations were largely replicated (Figure 1). To our knowledge, this is the first study that has evaluated how incorporating new populations influences the outcome of genomic offset analysis in a wild system. It is important to note, however, that genomic offset is a relative measure constrained by the populations included within a given analysis. As a result, we would expect the rank order of populations' relative values to ultimately change with the addition of a larger number of new populations. Such changes in genomic offset values could indeed be substantial in cases where new populations from previously unsampled regions are included (Lind and Lotterhos 2025); in this study, the populations that were added using GT‐seq were located within the kokanee range core. It remains to be tested whether including populations at the southernmost portion of the range in the USA or a higher relative proportion of unsampled populations would have a greater effect on these associations. Also, our analysis does not take into account the role adding new populations may play at the initial outlier detection step for identifying environment‐associated SNPs for panel inclusion; future work could test this by incorporating whole genome sequencing of the new populations to evaluate their relative influence on SNP selection and panel performance. Nevertheless, these results collectively suggest that GT‐seq can be effectively used to add more individuals and populations to existing baselines to inform assessments of genomic vulnerability to climate change at a fraction of the cost of WGS.

### 4.2 Donor/Recipient Importance Analysis

Across all different climate change scenarios and datasets used, DI values followed similar spatial trends, with populations currently situated in the warmest regions of BC, such as the Okanagan basin in the southern interior, having the highest probability for successful translocations to different recipient locations (Figure 3). These results are consistent with expectations, as current environments in the interior of BC are at the warmest end of the spectrum of conditions where kokanee persist, while at the same time being closer to future environmental conditions projected to occur in the coming decades for recipient locations elsewhere across the range. Likewise, the Sockeye Lake population in Yukon does not meet the threshold conditions for translocation to any other lake given the large disparity between current conditions at the northern range periphery relative to future projected conditions in potential recipient locations located to the south.

Recipient importance values also followed spatial trends: candidate lakes for stocking at the center of the distribution had higher RI values than those located towards the range periphery (Figure 3). This pattern is consistent with our DI results, as lakes at the center of the recipient range tended to be spatially closer to populations with higher DI; lakes towards the range peripheries showed the opposite pattern. Overall, these results indicate that geographical proximity is an important factor for determining DI and RI. Similar trends in DI/RI were observed in red spruce, where lower levels of DI were linked to local adaptation to more peripheral environments (Lachmuth, Capblancq, Keller, and Fitzpatrick 2023; Lachmuth, Capblancq, Prakash, et al. 2023), suggesting that this metric may be reflective of climate uniqueness for a given population. Moreover, RI can be interpreted as a measure of future habitat suitability, with higher values found in the current core ranges for both red spruce (Lachmuth, Capblancq, Keller, and Fitzpatrick 2023; Lachmuth, Capblancq, Prakash, et al. 2023) and kokanee (this study). Future applications to other systems will be necessary to more effectively generalize these patterns and further understand conditions under which spatial proximity may predict relative DI/RI.

### 4.3 Considerations for Fisheries Management

As highlighted in previous studies, further empirical research is needed to validate whether fitness declines with increasing offsets before integrating these metrics within a management context (Capblancq et al. 2020; Lachmuth, Capblancq, Keller, and Fitzpatrick 2023; Lind and Lotterhos 2025; Lotterhos 2024; Rellstab et al. 2021). Genomic offsets are often used as a proxy for fitness offset, with higher genomic offset values being understood to represent higher risks of maladaptation (Fitzpatrick et al. 2021). However, simulation studies have suggested that large genomic offsets can, in some cases, be indicative of a positive fitness offset (Lotterhos 2024). When identifying potential donor populations, establishing if high offset is representative of a positive or negative fitness impact is essential for informing management.

One main assumption of genomic offset studies is that populations are assumed to be currently adapted to their local environmental conditions (Fitzpatrick et al. 2021; Lotterhos 2024; Rellstab et al. 2021). However, salmonids have been shown to have varying degrees of local adaptation among populations (Fraser et al. 2011). In cases where populations are not adapted to their current environment, interpreting genomic offset values can be more challenging, and results need to be considered within their proper ecological contexts (Lotterhos 2024; Schmidt and Russello 2025). In this case, kokanee throughout British Columbia have exhibited vulnerability to increased water temperatures, with reports of populations undergoing increased summer kills due to extreme heat waves (Ward et al. 2019). These reports are consistent with warmest temperatures being the strongest predictor of environment‐associated genomic variation in this system (Tigano et al. 2024), as well as the strong correlation between warmest temperatures and higher genomic offset values demonstrated here. Taken together, these results provide indirect evidence that kokanee are locally adapted, while suggesting future maladaptation and vulnerability to increasing temperature levels projected by various climate change models. However, experimental validation of the fitness associations to genomic offset values is still required. In this context, ongoing physiological genomic studies of kokanee pairing thermal challenge experiments with gene expression analyses from several of the same sites for which we have genomic offset estimates (Mayer et al. in prep) will provide an excellent opportunity to investigate the degree to which genomic offset reflects actual fitness in this system.

Overall, the application of genomic offsets and DI/RI measures in management is still in its infancy, and the methods employed here require further refinement before they are used to inform potential fisheries decisions. For example, Gradient Forest is one of several approaches used for predicting genomic offsets, and in our case, it is the approach we considered most appropriate for our system. However, other methods in addition to Gradient Forest, including Risk‐Of‐Non‐Adaptedness, Latent Factor Mixed Models, or Redundancy Analysis, have been shown to perform differently depending on the system and scenarios tested (Capblancq et al. 2020; Fitzpatrick and Keller 2015; Lind and Lotterhos 2025; Rellstab et al. 2021).

Identifying alternative approaches for determining the genomic offset threshold for DI/RI also needs to be further explored. We used a threshold of one standard deviation of current spatial offset based on previous DI/RI studies (Lachmuth, Capblancq, Keller, and Fitzpatrick 2023; Lachmuth, Capblancq, Prakash, et al. 2023). Yet, the most appropriate threshold may vary according to the application (Lachmuth, Capblancq, Keller, and Fitzpatrick 2023; Lachmuth, Capblancq, Prakash, et al. 2023). For example, Lachmuth, Capblancq, Keller, and Fitzpatrick (2023) evaluated the impact of offset thresholds on DI and RI results, using red spruce as a case study. They found that different thresholds significantly impacted the magnitude of results, but spatial patterns remained consistent. While these findings suggest that using one standard deviation of current spatial offset may be appropriate as a threshold for an initial risk assessment, additional research is required to explore whether local offset and/or population/region‐specific values can provide further refinement of thresholds for informing DI/RI moving forward (Lachmuth, Capblancq, Keller, and Fitzpatrick 2023; Varas‐Myrik et al. 2024).

Other factors would need to be taken into account for application to fisheries management practices, including practical and biological considerations. Specific donor locations might be favored as sources of broodstock simply due to feasibility and logistics of sampling. In addition, for *O. nerka*, as well as other species with genetically distinguishable reproductive ecotypes (Russello et al. 2012), selecting donor populations will need to take into account life history, spawning timing, and habitat availability at the recipient location to increase chances of a successful translocation. As suggested in previous DI/RI studies (Lachmuth, Capblancq, Keller, and Fitzpatrick 2023; Lachmuth, Capblancq, Prakash, et al. 2023), using different subsets of the transferability matrix to identify best source populations that also incorporate realistic management parameters (e.g., feasibility of sampling) is essential for maximizing the effectiveness of any targeted recommendations. For kokanee, the stocks in Kalamalka Lake provide an excellent example that balances maximizing DI with life history and logistical considerations. This location in the southern interior of BC is home to both shore‐spawning and stream‐spawning kokanee, contains readily accessible spawning grounds for broodstock collection, and has a DI value approaching 95% for the recipient locations included in this study. Although further validation is necessary before integrating DI into stocking decisions, this example illustrates an approach that could be employed in the future.

Although genomic offset calculations and DI/RI models clearly need to be further refined, these methods hold great promise for informing freshwater fisheries management moving forward. Here, we have shown how pairing GT‐seq with measures such as Donor/Recipient Importance can help operationalize these new tools as part of a comprehensive kokanee fisheries management strategy.

## Conflicts of Interest

The authors declare no conflicts of interest.

## Supporting information


**Data S1:** eva70149‐sup‐0001‐Supinfo.docx.

## Data Availability

SNP genotypic data and associated R code are available in the Dryad Digital Repository (https://doi.org/10.5061/dryad.hdr7sqvwq).
