# Supplementary material for: Application of Genomic Offsets to Inform Freshwater Fisheries Management Under Climate Change
Source: Evol Appl. 2025 Aug 27;18(8):e70149. doi: 10.1111/eva.70149 (PMC12382357; doi:10.1111/eva.70149)
Supplement: Supplementary file 1 — Data S1: eva70149‐sup‐0001‐Supinfo.docx. [file EVA-18-e70149-s001.docx]

Supplementary Figures and Tables

| Dataset | Data Collection Method | *N_pop_* | *N_ind_* | *N_snp_* | Comments |
| --- | --- | --- | --- | --- | --- |
| WGS-1412 | WGS | 16 | 194 | 1412 | WGS genotypic data at 1412 environment-associated SNPs collected in Tigano et al. (2024) |
| WGS-616 | WGS | 16 | 194 | 616 | Subset of WGS-1412 comprised of 616 environment-associated SNPs collected in Tigano et al. (2024); same loci as GTseq-616 |
| GTseq-616 | GTseq | 16 | 199 | 616 | GTseq genotypic data at 616 environment-associated SNPs collected in this study for new individuals from the same 16 baseline populations as in Tigano et al. (2024) |
| Expanded-616 | WGS/GTseq | 19 | 241 | 616 | WGS-616 plus GTseq data collected in this study for 3 new wild stock populations |
| DRI-616 | WGS/GTseq | 19 | 435 | 616 | WGS-616 plus GTseq-616 plus GTseq data only from Expanded-616 for 3 new wild stock populations |

Supplementary Table 1: Composition of datasets used in this study.

Supplementary Table 2: Raw and Standardized genomic offset values for all four datasets used under the RCP 4.5 climate scenario.

| Location | WGS-1412 | | WGS-616 | | GTseq-616 | | Expanded-616 | |  |
| --- | --- | --- | --- | --- | --- | --- | --- | --- | --- |
|  | Raw | Standard-ized | Raw | Standard-ized | Raw | Standard-ized | Raw | Standard-ized | |
| Anderson | 0.01819 | 0.535 | 0.01746 | 0.562 | 0.01071 | 0.679 | 0.01647 | 0.811 | |
| Arctic | 0.02039 | 0.762 | 0.01879 | 0.721 | 0.00903 | 0.396 | 0.01399 | 0.486 | |
| Arrow | 0.02042 | 0.765 | 0.01942 | 0.797 | 0.01161 | 0.832 | 0.01650 | 0.816 | |
| Bonaparte | 0.02041 | 0.764 | 0.01846 | 0.682 | 0.00993 | 0.547 | 0.01354 | 0.426 | |
| Christina | 0.02217 | 0.945 | 0.02107 | 1.000 | 0.01257 | 1.000 | 0.01785 | 1.000 | |
| Cowichan | 0.01656 | 0.367 | 0.01490 | 0.253 | 0.00786 | 0.196 | 0.01057 | 0.036 | |
| EastBarriere | 0.01751 | 0.465 | 0.01708 | 0.516 | 0.00970 | 0.509 | 0.01517 | 0.641 | |
| Kalamalka | 0.02087 | 0.811 | 0.01983 | 0.847 | 0.01187 | 0.876 | 0.01681 | 0.856 | |
| Kootenay | 0.02055 | 0.779 | 0.01953 | 0.811 | 0.01173 | 0.852 | 0.01665 | 0.835 | |
| Nicola | 0.01880 | 0.598 | 0.01835 | 0.668 | 0.01030 | 0.610 | 0.01592 | 0.740 | |
| Okanagan | 0.02168 | 0.895 | 0.02061 | 0.940 | 0.01226 | 0.942 | 0.01746 | 0.943 | |
| Puntzi | 0.01547 | 0.255 | 0.01468 | 0.226 | 0.00842 | 0.292 | 0.01248 | 0.287 | |
| Shawningan | 0.01444 | 0.149 | 0.01279 | 0.000 | 0.00669 | 0.000 | 0.01029 | 0.000 | |
| Sockeye | 0.01290 | 0.000 | 0.01344 | 0.078 | 0.00890 | 0.373 | 0.01274 | 0.321 | |
| Tchesinkut | 0.02274 | 1.000 | 0.02104 | 0.992 | 0.01174 | 0.854 | 0.01656 | 0.824 | |
| Wood | 0.02111 | 0.836 | 0.02006 | 0.875 | 0.01200 | 0.898 | 0.01700 | 0.881 | |
| Alouette | / | / | / | / | / | / | 0.01844 | 0.560 | |
| Tatuk | / | / | / | / | / | / | 0.01209 | 0.236 | |
| Duncan | / | / | / | / | / | / | 0.01639 | 0.801 | |

Supplementary Table 3: Raw and Standardized genomic offset values for all four datasets used under the RCP 8.5 climate scenario.

| Location | WGS-1412 | | WGS-616 | | GTseq-616 | | Expanded-616 | |  |
| --- | --- | --- | --- | --- | --- | --- | --- | --- | --- |
|  | Raw | Standard-ized | Raw | Standard-ized | Raw | Standard-ized | Raw | Standard-ized | |
| Anderson | 0.02448 | 0.627 | 0.02343 | 0.652 | 0.01405 | 0.725 | 0.02119 | 0.828 | |
| Arctic | 0.02548 | 0.739 | 0.02361 | 0.671 | 0.01204 | 0.424 | 0.01814 | 0.481 | |
| Arrow | 0.02674 | 0.881 | 0.02543 | 0.858 | 0.01515 | 0.889 | 0.02152 | 0.866 | |
| Bonaparte | 0.02669 | 0.875 | 0.02443 | 0.756 | 0.01347 | 0.637 | 0.01855 | 0.529 | |
| Christina | 0.02822 | 1.047 | 0.02681 | 1.000 | 0.01595 | 1.000 | 0.02266 | 1.000 | |
| Cowichan | 0.02015 | 0.140 | 0.01832 | 0.125 | 0.00987 | 0.099 | 0.01337 | 0.000 | |
| EastBarriere | 0.02354 | 0.521 | 0.02281 | 0.589 | 0.01310 | 0.582 | 0.01998 | 0.691 | |
| Kalamalka | 0.02717 | 0.929 | 0.02582 | 0.899 | 0.01539 | 0.924 | 0.02182 | 0.900 | |
| Kootenay | 0.02688 | 0.896 | 0.02554 | 0.871 | 0.01527 | 0.906 | 0.02166 | 0.882 | |
| Nicola | 0.02489 | 0.673 | 0.02413 | 0.724 | 0.01373 | 0.676 | 0.02079 | 0.782 | |
| Okanagan | 0.02775 | 1.000 | 0.02638 | 0.956 | 0.01567 | 0.965 | 0.02229 | 0.953 | |
| Puntzi | 0.02143 | 0.285 | 0.02034 | 0.334 | 0.01188 | 0.401 | 0.01720 | 0.375 | |
| Shawningan | 0.01895 | 0.000 | 0.01707 | 0.000 | 0.00921 | 0.000 | 0.01386 | 0.000 | |
| Sockeye | 0.02017 | 0.142 | 0.01924 | 0.221 | 0.01156 | 0.352 | 0.01616 | 0.257 | |
| Tchesinkut | 0.02593 | 0.790 | 0.02407 | 0.719 | 0.01359 | 0.656 | 0.01911 | 0.592 | |
| Wood | 0.02741 | 0.957 | 0.02605 | 0.923 | 0.01552 | 0.943 | 0.02201 | 0.921 | |
| Alouette | / | / | / | / | / | / | 0.02412 | 0.586 | |
| Tatuk | / | / | / | / | / | / | 0.01653 | 0.299 | |
| Duncan | / | / | / | / | / | / | 0.02152 | 0.866 | |

Supplementary Table 4: Donor Importance values under the RCP 8.5 scenario.

| Location | Latitude | Longitude | Donor Importance |
| --- | --- | --- | --- |
| Anderson | 50.654 | -122.38922 | 81.034 |
| Arctic | 54.422 | -121.67877 | 68.966 |
| Arrow | 49.386 | -117.9908 | 82.759 |
| Bonaparte | 51.262 | -120.5584 | 96.552 |
| Christina | 49.162 | -118.27605 | 84.483 |
| Cowichan | 48.873 | -124.2632 | 70.690 |
| EastBarriere | 51.276 | -119.79919 | 94.828 |
| Kalamalka | 50.173 | -119.32713 | 94.828 |
| Kootenay | 49.516 | -116.81701 | 72.414 |
| Nicola | 50.154 | -120.56482 | 96.552 |
| Okanagan | 49.669 | -119.68313 | 94.828 |
| Puntzi | 52.195 | -124.03814 | 72.414 |
| Shawningan | 48.630 | -123.63768 | 68.966 |
| Sockeye | 60.505 | -137.62749 | 0.000 |
| Tchesinkut | 54.091 | -125.60007 | 50.000 |
| Wood | 50.082 | -119.38975 | 94.828 |
| Alouette | 49.334 | -122.4184 | 72.414 |
| Tatuk | 53.529 | -124.2311 | 68.966 |
| Duncan | 50.403 | -116.9697 | 79.310 |
|  |  |  |  |

Supplementary Table 5: Recipient Importance values under the RCP 8.5 scenario.

| Location | Latitude | Longitude | Recipient Importance |
| --- | --- | --- | --- |
| Beavertail | 49.980 | -125.50554 | 50.000 |
| Prospect | 48.521 | -123.4396 | 70.833 |
| Long | 49.209 | -124.02159 | 75.000 |
| Shelton | 49.129 | -124.32305 | 66.667 |
| Paska | 50.523 | -120.65985 | 87.500 |
| Stump | 50.332 | -120.40552 | 87.500 |
| Pillar | 50.602 | -119.64618 | 87.500 |
| Hallamore | 51.497 | -120.12948 | 87.500 |
| Peter Hope | 50.303 | -120.32582 | 87.500 |
| Monte | 50.490 | -119.83421 | 87.500 |
| Turquoise | 50.829 | -121.68601 | 91.667 |
| Paul | 50.736 | -120.15896 | 87.500 |
| Dutch | 51.648 | -120.05966 | 87.500 |
| Grave | 49.853 | -114.83347 | 45.833 |
| Rosen | 49.401 | -115.26182 | 50.000 |
| Deep | 49.264 | -115.34969 | 50.000 |
| Baynes | 49.230 | -115.22152 | 50.000 |
| Rockbluff | 49.900 | -115.64218 | 50.000 |
| Moyie(North) | 49.346 | -115.83101 | 50.000 |
| Monroe | 49.363 | -115.85995 | 50.000 |
| Mineral | 49.345 | -115.85121 | 50.000 |
| Moyie(North) | 49.265 | -115.84617 | 50.000 |
| Stoney(Hahas) | 49.749 | -115.81665 | 50.000 |
| Boundary | 49.007 | -116.88405 | 58.333 |
| Jim Smith | 49.482 | -115.84293 | 50.000 |
| Kootenay | 49.630 | -116.90306 | 58.333 |
| Milford | 50.013 | -116.92797 | 58.333 |
| Loon | 49.717 | -116.91459 | 58.333 |
| Box | 50.212 | -117.72684 | 62.500 |
| Bobbs | 51.795 | -120.97355 | 91.667 |
| Ten Miles | 53.072 | -122.45919 | 83.333 |
| Dugan | 52.170 | -121.91157 | 91.667 |
| Puntchesakut | 52.975 | -122.94722 | 79.167 |
| Deka | 51.609 | -120.85106 | 91.667 |
| Hathaway | 51.642 | -120.83665 | 91.667 |
| Sulphurous | 51.625 | -120.823 | 91.667 |
| McLeese | 52.388 | -122.2867 | 87.500 |
| Horse | 51.605 | -121.19482 | 91.667 |
| Till | 52.033 | -122.36113 | 87.500 |
| Bridge | 51.526 | -120.76808 | 91.667 |
| Chimney | 51.928 | -121.9804 | 91.667 |
| Green | 51.441 | -121.08771 | 91.667 |
| Timothy | 51.842 | -121.23804 | 91.667 |
| Dor | 51.872 | -121.00594 | 91.667 |
| Ruth | 51.831 | -121.03425 | 91.667 |
| Rimrock | 52.483 | -122.31677 | 87.500 |
| Tyee | 52.403 | -122.05877 | 87.500 |
| Big | 52.389 | -121.7878 | 91.667 |
| Co-Op | 54.188 | -125.43341 | 45.833 |
| Dunalter | 54.470 | -126.75302 | 33.333 |
| Purden | 53.911 | -121.95953 | 79.167 |
| Camp | 54.065 | -123.18024 | 75.000 |
| Eena | 54.054 | -123.02001 | 75.000 |
| Ness | 54.018 | -123.16668 | 75.000 |
| Otter | 49.549 | -120.75604 | 87.500 |
| Yellow | 49.335 | -119.76647 | 79.167 |
| Twin Lakes | 49.321 | -119.73017 | 79.167 |
| Alleyne | 49.911 | -120.56456 | 87.500 |


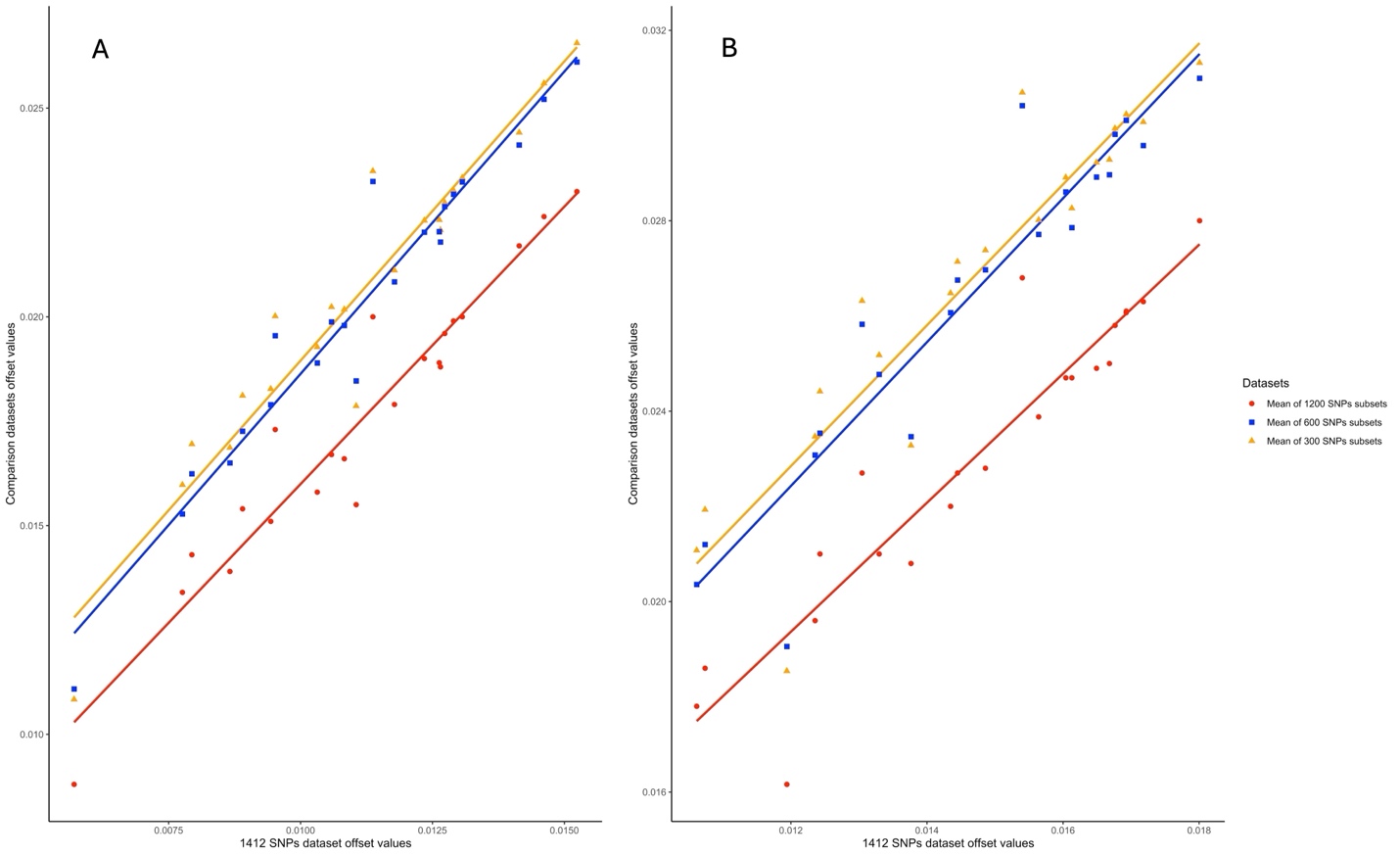


Supplementary Figure 1. Linear regression analysis of genomic offset values calculated from all random subsets of 1200, 600 and 300 SNPs relative to the WGS-1412 dataset for two different climate change scenarios including: A) RCP4.5 (mean r^2^ and p-values: SNP1200 x WGS1412, r^2^ = 0.999, p = 0.001; SNP600 x WGS1412, r^2^ = 0.974, p = 0.001; SNP300 x WGS1412, r^2^ = 0.922, p = 0.001); and B) RCP8.5 (mean r^2^ and p-values: SNP1200 x WGS1412, r^2^ = 0.999, p = 0.001; SNP600 x WGS1412, r^2^ = 0.982, p = 0.001; SNP300 x WGS1412, r^2^ = 0.954, p = 0.001.


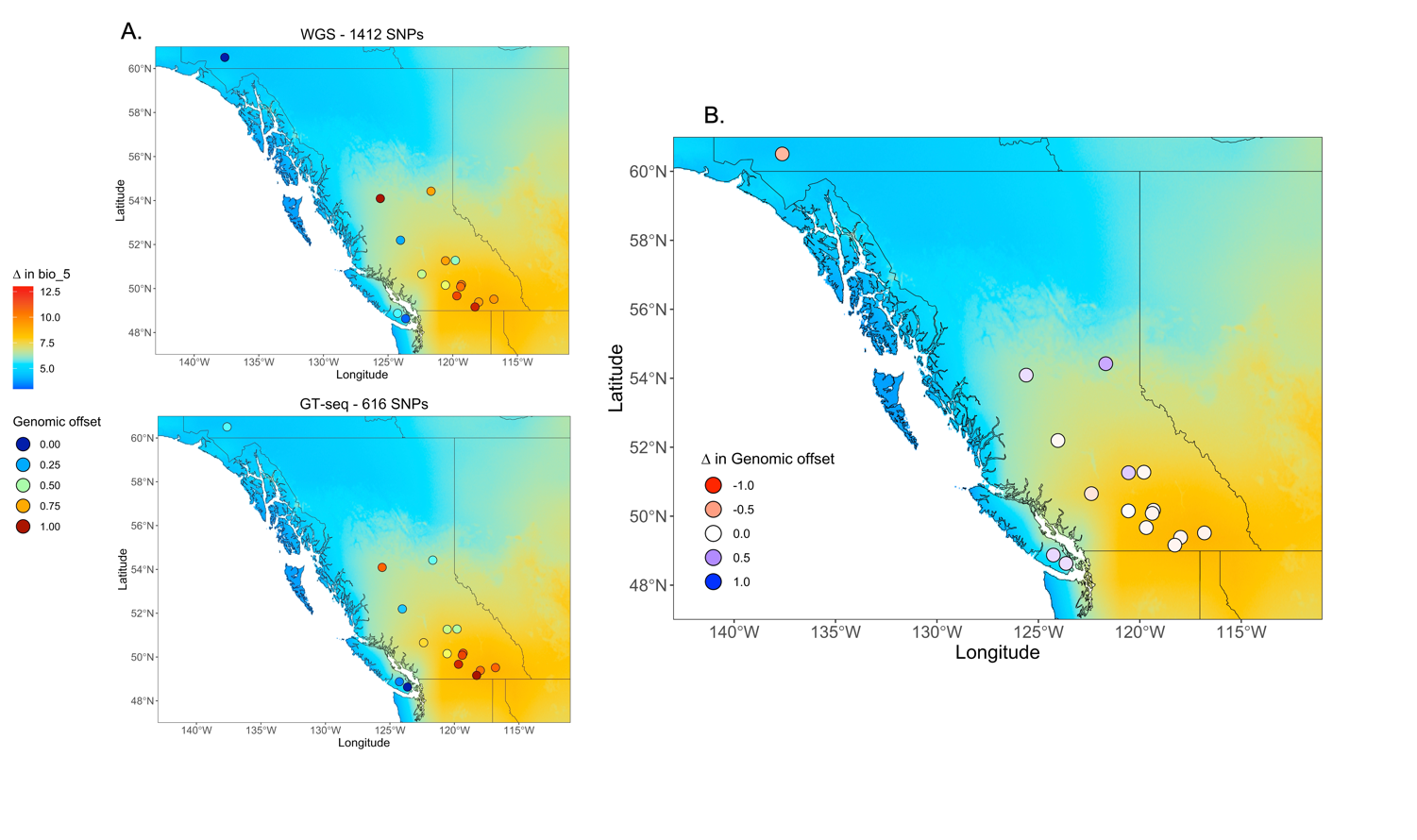


Supplementary Figure 2: Maps of the spatial distribution and relative difference of genomic offset values calculated from different datasets under the RCP4.5 climate change scenario. A). Spatial distribution of genomic offsets for the 16 wild stock lakes in BC and Yukon recalculated from the WGS dataset of Tigano et al. (2024) containing all 1412 environmentally-associated SNPs (WGS-1412; top) and based on GTseq data from new individuals genotyped from these same locations (GTseq-616). B). The relative change in genomic offsets between WGS-1412 and GTseq-616 at all 16 locations. The base map in all shows the difference in the warmest temperature of the of the warmest month (ΔT bio5) between current and predicted measures for 2041-2060 for the RCP4.5 climate change scenario
